# CNVizard—a lightweight streamlit application for an interactive analysis of copy number variants

**DOI:** 10.1186/s12859-024-06010-2

**Published:** 2024-12-17

**Authors:** Jeremias Krause, Carlos Classen, Daniela Dey, Eva Lausberg, Luise Kessler, Thomas Eggermann, Ingo Kurth, Matthias Begemann, Florian Kraft

**Affiliations:** Medical Faculty, Institute for Human Genetics and Genomic Medicine, Uniklinik RWTH Aachen, Pauwelsstrasse 30, 52074 Aachen, North-Rhine-Westphalia Germany

**Keywords:** CNV, NGS, CNVkit, AnnotSV, Snakemake, Long-read sequencing

## Abstract

**Background:**

Methods to call, analyze and visualize copy number variations (CNVs) from massive parallel sequencing data have been widely adopted in clinical practice and genetic research. To enable a streamlined analysis of CNV data, comprehensive annotations and good visualizations are indispensable. The ability to detect single exon CNVs is another important feature for genetic testing. Nonetheless, most available open-source tools come with limitations in at least one of these areas. One additional drawback is that available tools deliver data in an unstructured and static format which requires subsequent visualization and formatting efforts.

**Results:**

Here we present CNVizard, an interactive Streamlit app allowing a comprehensive visualization of CNVkit data. Furthermore, combining CNVizard with the CNVand pipeline allows the annotation and visualization of CNV or SV VCF files from any CNV caller.

**Conclusion:**

CNVizard, in combination with CNVand, enables the comprehensive and streamlined analysis of short- and long-read sequencing data and provide an intuitive webapp-like experience enabling an interactive visualization of CNV data.

**Supplementary Information:**

The online version contains supplementary material available at 10.1186/s12859-024-06010-2.

## Background

Copy Number Variations (CNVs), involving amplification or deletion of small or large segments of DNA [1], are a significant aspect of genomic variation. These variations contribute substantially to genetic diversity among individuals and populations, and they have been increasingly recognized for their role in the etiology of various genetic diseases [1, 2].

Historically large CNVs have been mostly analyzed using microarrays, while smaller CNVs have been targeted using multiplex ligation-dependent amplification (MLPA). In MLPA individual genes are analyzed using a probe mix which is highly specific. While legacy methods such as MLPA and microarray are still used, analysis of patients with genetic disease based on exome- and genome-wide massive parallel sequencing (MPS) has become the first line diagnostic approach in recent years. MPS data affords comprehensive CNV detection in addition to single nucleotide variants (SNVs) [3], and CNV analysis of MPS data has therefore started replacing legacy methods for CNV detection more and more. Accordingly, an increasing number of bioinformatic tools for CNVs analysis in MPS data are available (e. g. CNVkit [4], CNVnator [5], GATK [6]). Most of these tools are suitable for the identification of larger CNVs which comprise several exons of a gene or even larger parts of the genome. However, in genetic testing and research also single exon alterations must be identified reliably, as they can cause loss-of-function of the affected gene. Some of the available CNV analysis tools also allow the calling of single exon deletions or amplifications, e.g. CNVkit [4]. However, beside the calling of these variants a comprehensive visualization of CNV data is also important. Nevertheless, most tools lack a comprehensive visualization function, e.g. single exon MLPA/Coffalyzer-like CNV plots. This lack of visualization options might be hindering the transition from e.g. MLPA-based to MPS-based CNV analysis. Moreover, a comprehensive annotation of the data with known pathogenetic and database-curated CNVs is required to fosters a fast and reliable analysis. Though several tools are available for CNV calling and/or visualization, most of them lack some of the features, e.g. single exon or family-based analysis, necessary for comprehensive data analysis and visualization in genetic testing and research.

Here we describe CNVizard, a python tool featuring a browser-based graphical user interface created with Streamlit and a Snakemake [7] pipeline (CNVand [8]), to prepare the files such that they can be analyzed with CNVizard. The CNV analysis is based on CNVkit [4], which allows CNV calling of targeted and genome-wide data down to single exon level. Furthermore, CNVizard enables an interactive visualization. For data annotation we utilized AnnotSV [9], which enables a comparison with known pathogenic and benign CNVs.

## Implementation

CNVizard is developed in Python 3.12.4 and provides an interactive, browser-based environment implemented as a Streamlit application, enabling structured analysis of CNVs. CNVizard offers filterable data grids using pandas [10] and interactive plots generated with plotly [11] and seaborn [12, 13]. The tool features two modules: a companion module visualization of CNVkit [4] data and for exon-level filtering, and another for visualizing AnnotSV [9]-annotated variant call format (VCF) files.

CNVkit [4] is a Python package, and command-line tool designed to call CNVs from MPS data, with resolution down to the single-exon level. It belongs to a class of CNV calling algorithms that rely on read depth and B-allele frequency (BAF) strategies. In short, these algorithms predict CNVs by comparing the number of reads at specific locations to those in a reference dataset and by analyzing the data for abnormal B-allele frequency patterns.

AnnotSV [9] is a tool for annotating CNV data with additional information, supporting the interpretation of pathogenicity. Both tools are integrated with a Snakemake-based pipeline, CNVand [8], which processes data from BAM/CRAM files for visualization and analysis with CNVizard.

## Data input

### Data files

Using the Streamlit upload widget files can be uploaded to CNVizard via the web GUI. Depending on the required functionality, CNVizard is designed to work with formatted output provided by CNVkit [4] and AnnotSV [9]. The Snakemake [7] workflow provided along with CNVizard, CNVand [8], prepares all necessary files, starting from alignment files (BAM or CRAM). By providing the option to combine an exon-level resolution analysis for CNVs with the flexibility to additionally review annotated VCFs generated by different copy number callers, CNVizard enables an extensive analysis of CNVs.

In brief, CNVizard uses different outputs of CNVkit [4] the copy number regions (cnr) file, the bintest file which is a modified cnr file and the copy number segments (cns) file. The cnr file contains two values for the coverage depth. First, a bias corrected value called “log2 coverage depth” representing the comparison of the coverage depth of a region with defined size (bin) to the average coverage depth of a pooled reference, with outliers being removed. Second, a non-bias corrected coverage depth value called “depth” representing the mean coverage depth of the bin. Additionally, the cnr file contains a parameter “weight” which originates from the comparison of the bin size of each bin to the average bin size and binned reference log2 values. The bintest file additionally contains a p-value calculated by a binwise z-test which is corrected for multiple hypothesis testing. In contrast to the cnr and bintest file, the cns file contains the previously introduced values aggregated to larger regions which are called segments. Furthermore, the AnnotSV [9] TSV output is mandatory for visualization and filtering of CNV VCF files.

### Configuration files

Some CNVizard functionalities can be customized via configuration files. These include a tab-delimited text file utilized for formatting the AnnotSV [9] input data, allowing the user to choose the annotation that should be displayed in the CNVizard interface from among the comprehensive information provided by AnnotSV [9]. Moreover, an env file can be provided to enable Integrated Genomics Viewer (IGV) outlinks and text files with lists of genes, which enable a panel-based analysis. A new env file can be created directly from the Streamlit interface.

### Reference files

To obtain internal frequencies from internal exome or genome sequencing cohorts we concatenated cnr files using pandas [10] and subsequently calculated exon-level frequencies. The resulting reference file contains various frequencies (including frequency of heterozygous deletion frequency, frequency of homozygous deletion, and frequency of amplification), values necessary to create a boxplot (mean depth, mean log2, median depth, median log2, standard deviation of depth, standard deviation log2 and quartiles) and minimal and maximal log2 and depth values observed in the reference. We provide frequencies of our exome and genome cohorts as precomputed reference files. In addition, new references can be created from within the CNVizard application, using the scripts provided by the application and new data provided by the user.

## Core functionalities

### Individual exon-level CNV analysis

**Fig. 1:**
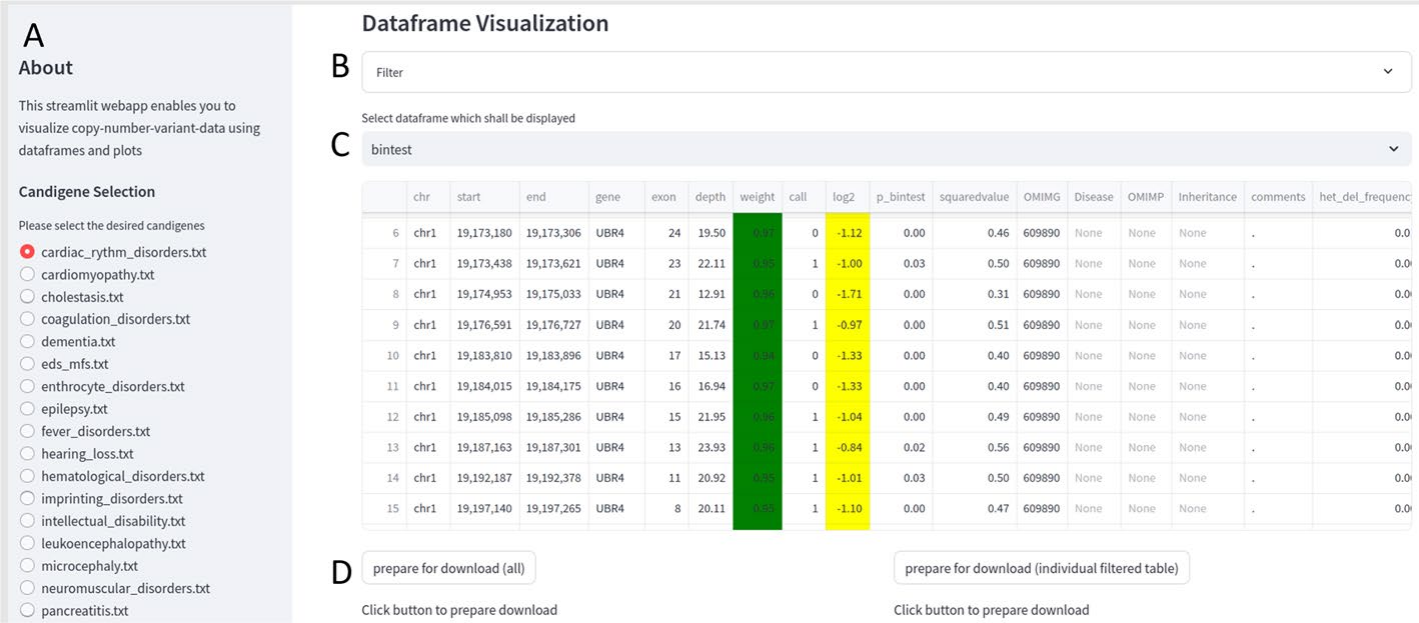
CNVizard web interface. On the sidebar a gene-panel list can be selected for a panel-based analysis. In this screenshot the preset “bintest” has been selected. A deletion of *UBR4* is detected (C, column call and log2). **A** Sidebar with gene-panel selection; **B** Filter-section: drop down menu which enables the user to interactively filter the data grid; regarding genomic region/gene, minimal read depth, copy number, minimal log2 ratio and database frequencies. **C** Interactive data grid with color-coding for CNVs (CNV 2 is shown in white, whereas CNV below—0.65 are marked in yellow); **D** Download button, which allows the downloading of the filtered or unfiltered data grid

Utilizing the copy number ratio (cnr) file and the output from the additionally performed bintest provided by CNVkit [4], pandas [10] is used to generate formatted data frames which can be filtered according to the user provided custom settings or according to seven provided presets, including “total” (represents a formatted version of the unfiltered cnr file), “bintest “ (represents a formatted version of the unfiltered bintest output file), “homozygous deletion” (data from the cnr file, filtered for homozygous deletions), “total candidate genes” (data from the cnr file, filtered for CNVs contained inside a candidate gene list), “bintest candidate genes” (data from the bintest output file, filtered with a candidate gene list), “consecutive deletions” and “consecutive amplifications” (data from the cnr file, filtered for consecutively deleted exons; the cut-off value can be set by the user). Custom settings can be applied for genomic regions/gene, minimal read depth, copy number, minimal log2 ratio and inhouse frequencies, in the panel above the results table, which enable the interactive modification of the results. All data grids also contain an internal frequency for each predicted CNV, calculated from an internal cohort. The scripts for frequency calculation are provided along with CNVizard and are also directly accessible from within the Streamlit interface. We provide several lists of candidate genes for different genetic conditions; additional ones can be added by the user. For this we provide a script which can be used to transform PanelApp (Genomics England [14]) TSV files into compatible TXT files. This functionality is also available from within the CNVizard application. The user can select the preferred gene-panel list inside the sidebar of the Streamlit webapp (Fig. 1A). Additionally, the user can filter the “total” preset with a variety of adjustable filters (e.g. “chromosome”,”position”,“gene” etc.). An overview of the web application can be seen in Fig. 1.

### Interactive exon-level MLPA/Coffalyzer-like log2 and raw depth boxplots

**Fig. 2:**
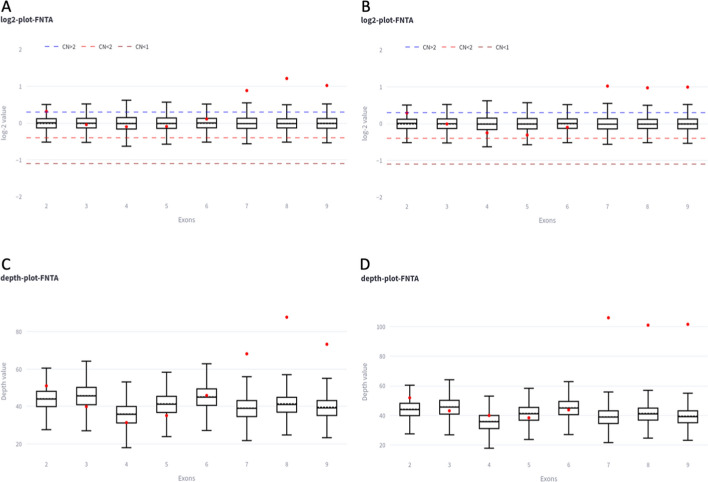
Examples of MLPA-like boxplots. The plots (A-D) show an example of a duplication of three exons within the *FNTA* gene. For exons 7, 8 and 9, the red dots, indicating the copy number or the coverage depth for the individual samples, are above the box plots, showing the copy number and coverage depth range of the reference samples. (upper panel: The blue and light red dashed line indicate the threshold for a copy number higher (0.3) or lower than 2 (−0.4). The red dashed line illustrates the threshold for a copy number below 1 (− 1.1). Upper panel/lower panel: Box plots indicate the 0.25 and 0.75 quartile of the reference samples. The dashed black lines indicate the mean, the solid black line the median. The whiskers show the minimum and maximum values of the reference samples, and the red dots indicate the copy number or depth of the analyzed single sample. A comparison between short-read data (**A** and **C**) and long-read data is shown (**B** and **D**). Most elements of the plot can be modified by the user ((log2 thresholds for duplications and deletions (indicated by the doted lines) and the color of the plotted elements)

For the exon-level CNV analysis we utilized these two metrics from the CNVkit cnr file, log2 coverage depth and depth, to generate MLPA/Coffalyser-like box plots by computing the median, mean, upper and low quartile of each exon from a pool of reference samples. These values are pre-calculated and allow the use of different datasets for exome and genome sequencing. Additionally, scripts are provided to create new reference files. With the data of the reference sample the user can create an exon-level box plot, by picking a gene (via an autocompletion input box), a plot reference, a sample log2 and raw depth values to analyze the copy number of the individual exons of a gene. The box plots are generated on-the-fly using plotly [11] and are adjustable by the user. In brief, the user can customize the log2 thresholds for duplications and deletions (indicated by the doted lines, default duplication (0.3), heterozygous (-0.4) and homozygous deletion (-1.1)), and the color of the different elements of the plot. Examples of coverage plots can be seen in Fig. 2.

### Trio mode

CNVizard also offers an option to provide parental samples which enable a trio analysis, e.g. to filter for de novo variants. This is achieved by preprocessing the samples using CNVkit, and subsequently CNVizard performs a left join on the index and parental cnr files.

### Genome-wide and chromosome-wide scatter plot

**Fig. 3:**
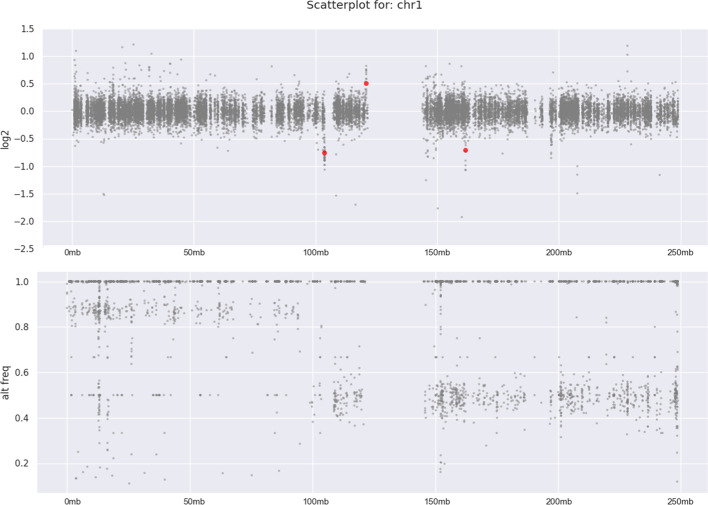
CNV and b-allele frequency scatter plot as provided by CNVizard for chromosome 1. A somatic loss of heterozygosity is visible, indicated by the dispersion of the grey dots towards 1 and 0 in the B-allele frequency plot (B) on the left side (p arm) compared to the normal bi-allelic state on the right side of the plot (q arm). The CNV plot shows three small CNVkit-called copy number alterations (red dots in the CNV plot (A)). Grey dots indicate the copy number of a single bin as analyzed by CNVkit [4]. Regions with copy number changes called by CNVkit, are indicated by red dots. The x-axis depicts the chromosomal position in Mb and the y-axis, either the log2 copy number or the allele frequency ratio. Plots are generated using matplotlib [12] and seaborn [13]. The user can modify the displayed region (either the whole genome or a single chromosome) and the color of dots for copy number changes

Similar to CNVkit [4], CNVizard can create scatter plots for log2 copy number values and B-allele frequency plots, based on the CNVkit [4] cnr and cns files. The log2 copy number plot shows the log2 value of every bin in the cnr file and additionally highlights segments with a copy number alteration, called by CNVkit [4]. Furthermore, if a VCF file is provided by the user, a B-allele frequency plot is generated. This plot shows the allelic balance of SNVs from the VCF file, which can be either 0 (both alleles are reference), ~ 0.5 (one allele is reference and the other an alternative call) or 1 (both alleles are an alternative call). Alterations in the allelic distribution of variants are called by CNVkit [4] and highlighted in the scatter plot. We integrated this functionality into CNVizard, to allow the user to plot and subsequently analyze CNV data on chromosome or genome level, which can be chosen by a drop-down menu. Moreover, loss of heterozygosity (LOH) can be analyzed using the B-allele frequency plot. An example of a chromosomal scatter plot can be seen in Fig. 3. Consecutive regions with only homozygous SNV calls, either 0 or 1, indicate a LOH.

## CNV annotation and prioritization

Analyzing CNVs for pathogenicity requires an extensive annotation. Therefore, within a second module of CNVizard, we implemented the support for AnnotSV [9] annotated VCF files, which could be either generated by the CNVand [8] pipeline or any other workflow. AnnotSV [9] is compatible with the majority of CNV callers which provide their output in form of a VCF or BED file. Hence, the second module of CNVizard could also be used also with data from other CNV callers, providing an adjustable and interactive data grid of CNV VCF records. CNVizard allows to reduce the comprehensive annotation of AnnotSV [9] with a configuration file, which contain all columns which should be included into the interactive data grid in CNVizard. Furthermore, CNVizard provides several options to filter the data in the interactive table.

Using the CNVand [8] pipeline for preparation of data for the CNV VCF visualization module, CNVizard combines the detection of exon-level CNVs using the CNVkit [4] bintest script and of larger CNVs, called with the CNVkit [4] standard workflow. This functionality complements the more targeted analysis and enables a “discovery mode” for all CNVs in a provided dataset.

## Snakemake-pipeline

Next to CNVizard we provide a Snakemake [7] pipeline which implements preprocessing steps, the CNV calling with CNVkit [4] and the annotation process with AnnotSV [9], starting from alignment and VCF files. In a first step the alignment files are sorted and indexed using Samtools [15]. Subsequently CNVkit [4] is used. In brief, the coverage is calculated for target and antitarget regions and a copy number reference is created. The resulting reference is used to generate the individual cnr files. Segmentation is performed utilizing the depth and BAF. Circular binary segmentation is used as default model for segmentation. CNVkit [4] provides additional models for segmentation which can be selected using a config for CNVand [8]. Subsequently the calculated log2 coverage depth values are translated into copy number calls, using the call function from CNVkit [4]. Additionally, for the identification of single-bin copy number alterations a z-test corrected with the Benjamini-Hochberg [16] method is performed (bintest provided by CNVkit [4]). At last, the CNV calls are exported as a VCF file, which is subsequently annotated using AnnotSV [9]. AnnotSV [9] is run in full annotation mode, the output is written as tab-separated-values file. CNVand [9] is compatible with panel, exome and genome sequencing data. The Snakemake [7] pipeline is available through Snakemake [7]-workflows and GitHub.

## Results

Whereas tools for CNV calling and visualization have been developed and published previously, to our knowledge neither of them combines the capabilities to analyze CNVs ranging from single exon resolution up to whole genome resolution in a streamlined process. We created CNVizard to address and improve upon these issues. To assess the usefulness of CNVizard, we compared it to similar already available open-source tools (these being CNspector [17], reconCNV [18], CNViz [19], Genomecat [20] and knotAnnotSV [21]), with respect to various aspects that are important for a streamlined CNV analysis (Table 1). The first criterion is the ability to robustly call CNVs, therefore enabling an analysis workflow independent of an existing pipeline. Along with 3 out of 5 other tools (CNspector [17], reconCNV [18], GenomeCAT [20]) CNVizard can perform independent CNV calling with the CNVand [8] Snakemake [7] pipeline. Whereas CNspector [17] implements their own CNV calling algorithm, CNVizard utilizes the widely used and actively maintained tool CNVkit [4] for CNV calling via CNVand [8].

**Table 1:** Side by side comparison of CNVizard towards similar open source CNV visualization applications

Functionality provided	CNspector (17)	reconCNV(18)	CNViz(19)	GenomeCAT(20)	knotAnnotSV(21)	CNVizard
CNV-calling	Yes	Yes	No	Yes	No	Yes
Interactive datagrid	Yes	Limited	Limited	Limited	Extensive	Extensive
Scatterplot (genomewide)	Yes	Yes	Yes	Yes	No	Yes
Boxplot (gene/exon-based)	No	No	No	No	No	Yes
Annotation	Depends on input data	Limited	Limited	Limited	Extensive	Extensive
OMIM-integration	No	No	No	No	Yes	Yes
Panel based analysis	Yes	No	No	No	No	Yes
Loss of heterozygosity	Yes	No	Depends on input data	No	No	Yes
Single-exon resolution	Limited	No	No	No	Depends on input data	Extensive
Family / trio mode	Yes	No	No	Yes	No	Yes
Compatibility with third party CNV-Callers	Yes	Yes	Yes	Yes	Yes	Yes
Architecture	R / Shiny App	Python / env	R / Shiny App	Java / Installation Wizard	Perl	Python / env / streamlit

A second important criterion is the presentation of CNV data in an interactive data grid. While all tools provide a data grid in some form, only knotAnnotSV [21] and CNVizard provide filter options to customize the data grid. Both tools provide flexible filtering options and contain sufficient annotations presented in a structured format.

The third criterion is the data visualization of CNVs using interactive plots. While most tools (4 out of 5, CNspector [17], reconCNV [18], CNViz [19], GenomeCAT [20]) have the option to analyze the ingested data for larger structural alterations using a scatter plot, only CNVizard enables plotting for smaller gene/exon-level alterations.

The fourth criterion is the support for different annotation resources. The majority of previously published tools (4 out of 5, CNspector [17], reconCNV [18], CNViz [19], GenomeCAT [20]) provide only sparse annotations for CNVs. Additionally, some of them utilize integrated annotation sources, which are susceptible to be outdated, if not properly maintained. By providing support for AnnotSV [9], which is a widely used and actively maintained framework for the annotation of CNVs, CNVizard can provide an exhaustive number of annotations for larger CNVs and supports a more condensed number of annotations (inhouse frequency, OMIM-annotations and Inheritance) for smaller CNVs. The importance of up-to-date resources for annotation have been already demonstrated [22].

The fifth criterion is the capability to support a panel analysis. Depending on the type of genetic testing or research focus, only a few genes may be of interest for the analysis. Gene panels are only implemented by the minority of previously published tools or require a reformatting of the input data (1 of 5, CNspector [17]). To overcome this limitation, CNVizard has a straightforward easily adjustable implementation of gene panels.

Furthermore, we compared the tools for their capability of performing an analysis for loss-of-heterozygosity. We implemented this feature in the CNVizard using genome wide B-allele frequency plot, which can aid in the analysis of somatic CNVs and uniparental disomies (UPD). Next to CNVizard, 2 out of 5 tools (CNspector [17] and CNViz [19]) also provide this feature.

One of the important features of CNVizard is the ability to analyze single exon CNVs. To achieve this CNVizard provides a high resolution CNV analysis in the form of an interactive data grid, Furthermore, CNVizard offers box plots for CNV and sequencing depth in single exons-resolution, similarly to MLPA analysis. (Fig. 2). Additionally, CNVizard provides internal frequencies for single-exon CNVs, enabling further filtering and prioritization. To our knowledge no other tool provides such a high resolution for single exon analysis, yet there are numerous examples in the literature demonstrating that single exon CNVs are a vital source of genetic disorders and that they are often missed by other CNV analysis approaches [22–25].

Genetic testing and research often involve family-based studies, such as trio analysis, where the data of an affected individual is analyzed in conjunction with their parents' data. CNVizard provides a “family mode” to allow the discovery of de novo CNVs, which is only supported 2 out of 5 comparable tools (CNspector [17], GenomeCAT [20]).

Due to the option to use VCF files as data input, all tools, including CNVizard are also compatible with other CNV callers. By relying on AnnotSV [9] as an annotation tool, which is compatible with a variety of different CNV-calling algorithms, CNVizard inherits this compatibility in the context of VCF files. However, using VCF files as input limits the functionality of most of the tools. In case of CNVizard, only the second module, which provides annotation and filtering of CNV data in an interactive data grid is compatible with VCF input files. The comprehensive analysis of single-exon CNVs and the trio-analysis are only available using BAM/CRAM files.

CNVizard is easy to set up, as it is open source and available via GitHub or pypi. The tool is provided as a python package, which installs all dependencies automatically. Alternatively, a dockerfile is provided as well as continuous integration for the GitHub releases. Its unique feature is the comprehensive implementation of a CNV analysis environment which offers a high-resolution analysis of CNVs, which is a relevant topic in the research of monogenetic diseases. CNVizard offers a pipeline for CNV calling (CNVand [8]), starting from alignment files. Its user interface provides an interactive data grid with various filter options, to allow the analysis and visualization of single exon CNVs similar to MLPA/Coffalyser analysis. Furthermore, it provides a comprehensive configurable annotation via AnnotSV, in addition to gene panel-based filter strategies and trio analysis. Finally, parts of CNVizards functionality are compatible with other CNV callers, besides CNVkit [4].

In summary, CNVizard is a lightweight CNV analysis toolkit which enables a comprehensive analysis of CNV data for diagnostic and research applications.

## Supplementary Information

Additional file 1.

## Data Availability

All code required to setup CNVand (https://github.com/IHGGM-Aachen/CNVand) and CNVizard (https://github.com/IHGGM-Aachen/CNVizard) is available on Github. Additionally CNVand is available on WorkflowHub. CNVizard can also be installed using pypi. Operating systems: Ubuntu, MacOS and also available in a Docker Container. Programming Language: Python. Other Requirements: Python 3.12.4 or higher, Tabix/Samtools 1.21 or higher. License: MIT License. Any restrictions to use by non-academics: None.

## Abbreviations

BAF: B-allele frequency
cnr: Copy number regions
cns: Copy number segments
CNV: Copy number variant
IGV: Integrated genomics viewer
MPS: Massive parallel sequencing
MLPA: Multiplex ligation dependent probe amplification
NGS: Next generation sequencing
SNV: Single nucleotide variant
VCF: Variant call format
