# Supplementary material for: CNVizard—a lightweight streamlit application for an interactive analysis of copy number variants
Source: BMC Bioinformatics. 2024 Dec 17;25:376. doi: 10.1186/s12859-024-06010-2 (PMC11650836; doi:10.1186/s12859-024-06010-2)
Supplement: Supplementary file 1 — Additional file 1. [file 12859_2024_6010_MOESM1_ESM.docx]

**Supplement**


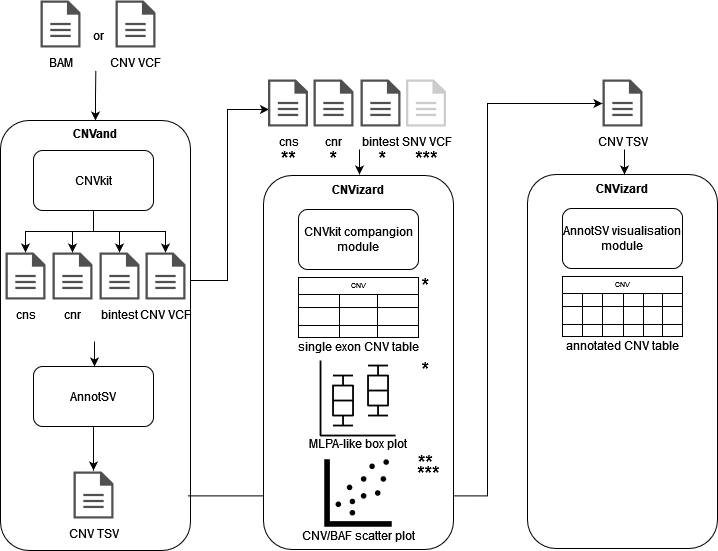


Suppl. Fig. 1 Input requirements and output of CNVizard and CNVand

The required input files for CNVizard vary depending on the module being used. For the CNVkit companion module, at least the cnr and bintest files are needed to create a single exon CNV data grid and MLPA-like box plots. If a CNV scatter plot is also desired, the cns file is mandatory. Additionally, generating a B-allele frequency plot requires a VCF file containing SNV data. Three of the four input files (cnr, cns, and bintest) can be produced either via the CNVand pipeline or directly using CNVkit.

For the AnnotSV visualization module, only an AnnotSV-annotated TSV file containing the CNV information is needed. This TSV file can be generated either using the CNVand pipeline or AnnotSV directly.
